# Review on colonization of residents and staff in Italian long-term care facilities by multidrug-resistant bacteria compared with other European countries

**DOI:** 10.1186/s13756-016-0136-1

**Published:** 2016-10-11

**Authors:** Richard Aschbacher, Elisabetta Pagani, Massimo Confalonieri, Claudio Farina, Paolo Fazii, Francesco Luzzaro, Pier Giorgio Montanera, Aurora Piazza, Laura Pagani

**Affiliations:** 1 Laboratorio Aziendale di Microbiologia e Virologia, Comprensorio Sanitario di Bolzano, Bolzano, Italy; 2 U.O. di Microbiologia, Azienda Sanitaria Locale di Piacenza, Piacenza, Italy; 3 Azienda Socio-Sanitaria Territoriale Papa Giovanni XXIII, USC Microbiologia e Virologia, Bergamo, Italy; 4 P.O. Spirito Santo Laboratorio Analisi, Pescara, Italy; 5 Ospedale A. Manzoni, Laboratorio Microbiologia e Virologia, Lecco, Italy; 6 USL Valle D’Aosta, SC Laboratorio di Microbiologia, Aosta, Italy; 7 Dipartimento SCCDP, Unità di Microbiologia e Microbiologia clinica, Pavia, Italy

**Keywords:** LTCF, ESBL, MRSA, Carbapenemase, Colonization

## Abstract

**Background:**

Rates of colonization and infection with multidrug-resistant (MDR) bacteria are increasing worldwide, in both acute care hospitals and long-term care facilities (LTCFs). Italy has one of the highest prevalence of MDR bacteria in European countries, especially with regard to methicillin-resistant *Staphylococcus aureus* (MRSA) and extended-spectrum β-lactamase (ESBL) or carbapenemase producing *Enterobacteriaceae* (CPE).

**Method:**

Review of studies on colonization by MDR bacteria from Italian LTCFs, risk factors for colonization and molecular characteristics of surveillance and clinical isolates, compared with other European countries.

**Results:**

High variability of MDR colonization has been reported within and especially between European countries. Only a few surveillance studies have been performed in Italian LTCFs; these show MRSA colonization prevalence of 7.8–38.7 % for residents and 5.2–7.0 % for staff members, ESBL prevalence of 49.0–64.0 % for residents and 5.2–14.5 % for staff and prevalence of CPE of 1.0–6.3 % for residents and 0.0–1.5 % for staff. In Italian LTCFs, as well as in other European countries, the most prevalent ESBLs from surveillance or clinical *Escherichia coli* isolates were found to be CTX-M-type enzymes, particularly CTX-M-15, expressed by the pandemic ST131 clonal group; this lineage also expresses carbapenemase genes of the *bla*_VIM_ and *bla*_KPC_ types. Various risk factors for colonization of residents by MDR bacteria were identified.

**Conclusions:**

The limited data from Italian LTCFs confirms these settings as important reservoirs for MDR organisms, allowing important considerations regarding the infection risk by these organisms. Nevertheless, more extended and countrywide screening studies for MDR colonization in Italian LTCFs are required. To promote further studies of various microbiological aspects related to LTCFs, the Association of Italian Clinical Microbiologists (Associazione Microbiologi Clinici Italiani; AMCLI) in 2016 has set up a new Working Group for the Study of Infections in LTCFs (Gruppo di Lavoro per lo Studio delle Infezioni nelle Residenze Sanitarie Assistite e Strutture Territoriali assimilabili; GLISTer), consisting of Clinical Microbiologists represented by the authors of this review article.

## Background

As in other European countries the aging population in Italy represents an increasing public health priority. Long-term care facilities (LTCF), according to the definition used in this review article, are facilities (nursing homes, inpatient behavioral health facilities, and long-term chronic care hospitals) that help meet both the medical and non-medical needs of elderly people with a chronic illness or disability, involving various levels of medical care that require the expertise of skilled practitioners. The older, sicker residents cared for in LTCFs have a variety of risk factors for colonization and also for infection with multidrug-resistant (MDR) organisms, increasing their morbidity and mortality [1]. Antibiotics are among the most commonly prescribed classes of medications for LTCF residents, increasing the selection pressure for MDR bacteria [2]. Cross-transmission within these facilities is also promoted, owing to various factors such as permanent living in a confined environment, the difficulty of diagnosing infections that present atypically and because of the frequent cognitive impairment of residents, reducing the compliance with basic hygiene measures. For these and other reasons LTCFs are a potentially important reservoir for MDR organisms [1, 3].

The aim of this review is to summarize the colonization prevalence studies of LTCF residents and staff published from Italian LTCFs and to compare them with studies from other European countries. MDR bacteria considered in this review are methicillin-resistant *Staphylococcus aureus* (MRSA), vancomycin resistant enterococci (VRE), extended-spectrum β-lactamase (ESBL) and/or carbapenemase producing *Enterobacteriaceae* (CPE). Furthermore, the main bacterial genotypes and resistance genes circulating in Italian LTCFs are reviewed. Finally, major risk factors for colonization by MDR bacteria identified in Italian LTCFs, compared with other European countries, are discussed.

### Literature search

We adopted a search strategy in the Medline/Pubmed database including the following search terms: (nursing home* OR long term care facilit*) AND (colonization OR multi drug resistan* OR ESBL OR MRSA OR VRE OR carbapenemase*). We restricted the search to the date range 01.01.2000–30.08.2016 but did not impose any language restriction. Applying this search strategy we identified 641 articles and retrieved 14 full-text Italian and 68 full-text European non-Italian studies. Moreover, we included a poster referred to Italian LTCFs (reporting colonization prevalence and typing results), presented at the 26^th^ European Congress of Clinical Microbiology and Infectious Diseases (ECCMID), in the reference list. Overall, we included 28 LTCF colonization studies from European non-Italian countries in the review; only 2 studies investigated carriage with CPE and 4 studies assessed the colonization by VRE, while 20 studies described MRSA carriage and 12 studies reported colonization by ESBL producers. Furthermore, we included 6 colonization studies from Italy, comprising 5 studies reporting MRSA, 4 studies describing ESBL, 3 studies investigating CPE and only 1 study referred to colonization by VRE. Eleven studies from the Medline/Pubmed search reported molecular characterization of Italian LTCF isolates (comprising one study reporting typing of MRSA); from the reference lists of the articles we retrieved two further Italian studies reporting molecular characterization data of isolates. Exclusion criteria for reviewing of colonization rates were studies that selected for clinical isolates or were referred to outbreak investigations, whereas similar studies were included in the analysis of resistance gene types and in typing of isolates; a structured review of the molecular characterization of isolates was done only for Italian LTCF isolates. At least two authors screened every abstract and full-text article and they discussed the results with all other authors before they were included in the review article.

### Colonization of residents and staff by MDR bacteria in European LTCFs

Rates of colonization and infection with MDR bacteria are increasing worldwide, in both acute care hospitals and LTCFs [1–3]. Colonization rates by MRSA and ESBL producing *Enterobacteriaceae* in European LTCFs are summarized in Table 1; the surveys are quite heterogeneous in terms of facility sizes, resident characteristics and specimen types and therefore direct comparison between different studies is subject to bias. Nevertheless, high variability of colonization frequency for ESBL producing *Enterobacteriaceae* and MRSA, ranging from close to zero up to levels higher than 50 % can be derived from these studies; variability is high especially among countries but also among different LTCFs within single countries. Recently, the emergence and worldwide spread of CPE has generated an immediate infection threat to residents in LTCFs, but we found only two European colonization studies reporting low colonization prevalence; 0.0 % in a study from Ireland [4] and 0.3 % in a study from the Netherlands [5]. Colonization by VRE in European LTCF residents was found to be low, ranging from 0.0 % in Belgium [6], 0.4 % [7] and 2.7 % [8] in Germany, up to 3 % in Ireland [4]. LTCF staff colonization by MRSA in European LTCFs was found as high as 7.5 % in Ireland [9] and 0.0–5.2 % in Slovenia [10].

**Table 1:** Colonization of European LTCF residents with MRSA and ESBL producing *Enterobacteriaceae*

Country, year	Residents/LTCFs (number)	Specimen type	MRSA % (range %)	ESBL % (range %)	References^a^
Sweden, 2008	495/9	Rectal	0.0	3.0	[54]^b^
Sweden, 2010	163/1	Catheter Urine		0.6	[55]
Sweden, 2014	91/10	Rectal		11	[56]^b^
Netherlands, 2009	1268/12	Nasal	0.3		[57]
Netherlands, 2012–14	4763 (MRSA), 5421 (ESBL)/107	Nasal (MRSA) Urine (ESBL, *E. coli*)	0.3	2.2	[51]
Germany, 2009	1827/32	Nasal and/or wound	7.6 (0–18.6)		[45]
Germany, 2010–11	240/11	Rectal		9.6 (0–30)	[18]
Germany, 2012	150/8	MRSA: nasal, pharyngeal ESBL: perineal	9.2	26.7	[8]
Germany, 2013	690/26 (MRSA), 455/26 (ESBL)	Nasal, pharyngeal, perineal	6.5	17.8	[7]
Germany, 2013–14	2858/65	Nasal, pharyngeal	4.8 (0–26.7)		[46]
Germany, 2013–14	156/31	Rectal		14.7	[19]
Luxembourg, 2010	954/19	Nasal, pharyngeal	7.2 (0–16)		[49]
Slovenia, 2001–02	107/1	Nasal and skin	9.3		[10]
France, 2004	109/1	Nasal, rectal	37.6		[52]
France, 2013	1155/38	Rectal or urine		9.9 (0.0–28.6)	[58]
Belgium, 2005	2953/60	Nasal, pharyngeal, wound, urine	19.5 (2.0–42.9)		[47]
Belgium, 2011	2791/60	Nasal, pharyngeal, wound, rectal	12.2 (0–36)	6.2 (0–20)	[6]
Spain, 2003	413/4	Nasal, skin	22.5 (17.2–35.5)		[59]
Spain, 2005	1377/9	Nasal	16.8 (6.7–35.8)		[60]
Spain, 2009–10	744/17	Nasal	19.6		[61]
UK, 2005	715/39	Nasal	22.2 (0–50)		[42]
UK, 2005–07	3037/69	Nasal	20 (19–21)		[43]
UK, 2006–09	2492/65	Nasal	20.5		[44]
Ireland, 2004–06	294/16	Rectal (only *E. coli*)		40.5 (0–75)	[17]
Ireland, 2005–06	1111/45	Nasal	23.3 (0–73)		[9]
Ireland, 2012–13	64/1	Nasal, rectal, urine	27	55	[4]^b^

^a^All studies are point prevalence studies, except three (^b^) that follow residents over three months [56], two years [54] or one year [4], respectively

Italy has one of the highest prevalence of MDR bacteria isolated from blood cultures in European countries, especially with regard to MRSA and ESBL- or carbapenemase-producing *Enterobacteriaceae* [11] and residence in a LTCF is an independent risk factor for MDR blood stream infections [12]. In spite of the importance of LTCFs as reservoirs for MDR organisms, in Italian LTCFs only a few colonization studies have been performed during the last 10 years (Table 2). These surveys show MRSA colonization prevalence of 7.8–38.7 % for residents and 5.2–7.0 % for staff, ESBL prevalence of 49.0–64.0 % for residents and 5.2–14.5 % for staff, and CPE prevalence of 1.0–6.3 % for residents and 0.0–1.5 % for staff. Colonization by VRE is rare, with 2.7 % of residents found in one study [13].

**Table 2:** Colonization of Italian LTCF residents and staff with MRSA and multi-drug resistant *Enterobacteriaceae*

Province, year	No. of residents (R), staff (S), LTCFs (L) included	Mean age in years/female %	Specimen types	Residents, colonizationwith MDR (%)	Staff, colonization with MDR (%)	References^a^
Bergamo, 2005	R (88), S (51), L (1)	83/80	Nasal	MRSA (19.3)	MRSA (5.8)	[39]
Vicenza, 2006	R (551), L (2)	83/73	Nasal	MRSA (7.8)		[62]
Various provinces^b^, 2006	R (221), L (23)	83/61	Catheter urine	ESBL/AmpC (54.0)		[22]
Bolzano, 2008^c^	R (111), S (69), L (1)	84/55	Rectal, inguinal, oro-pharyngeal, nasal, urine	ESBL (64.0) AmpC (4.5) Carbapenemase VIM-1 (6.3) MRSA (38.7) VRE (2.7)	ESBL (14.5) AmpC (1.5) Carbapenemase VIM (1.5) MRSA (14.5)	[13]
Bolzano, 2012^c^	R (131), S (57), L (2)	83/60	Rectal, inguinal, oro-pharyngeal, nasal, urine	ESBL (49.0) AmpC (2.9) Carbapenemase VIM-1 (1.5) MRSA (13.2)	ESBL (5.2) AmpC (0.0) Carbapenemase VIM (0.0) MRSA (7.0)	[14]
Various provinces^d^, 2015	R (489), L (12)	85/69	Rectal, nasal, axillary	ESBL (57.3) Carbapenemase KPC-3/VIM-1 (1.0) MRSA (17.2)		[23]

^a^All studies are point prevalence studies; ^b^ 70 % Northern Italy, 5 % Central Italy, 25 % Southern Italy; ^c^ one of the LTCFs was screened in 2008 and 2012; carbapenemase screening was done on chromogenic ESBL agar plates; ^d^ 50 % Northern Italy, 50 % Central Italy

All Italian and all except three European studies included in the review are point prevalence studies (Tables 1 and 2). Unfortunately, in many of these colonization studies, the only sample type for MRSA screening were nasal swabs, but by using exclusively this specimen type screening sensitivities of only 50–60 % are reached, with possible significant underestimation of the real colonization frequency; on the other hand the only use of rectal swabs for ESBL screening, as done in most of the colonization studies, permits still high sensitivity of 77–96 % [13, 14]. Screening for CPE in two of the three Italian colonization studies was done on chromogenic ESBL agar plates [13, 14]; this non-specific carbapenemase screening could have impacted on the sensitivity of the method. Finally, Italian colonization data refers mainly to a limited number of LTCFs, located in northern provinces of the country; because it is not appropriate to extrapolate data from Northern Italy to the central and southern part of the country, more extended and countrywide colonization studies are required.

### Molecular characterization of MDR *Enterobacteriaceae* and MRSA from surveillance or clinical samples of Italian LTCFs

ESBL producing *Escherichia coli* isolates frequently belong to the pandemic clonal-group ST131 (mainly the H30-ST131 sub-clone) [15]; the association of this lineage with LTCFs has been widely documented [16]. In European LTCFs the clonal *E. coli* ST131 group frequently expresses ESBL genes belonging to various CTX-M-types (prevalently CTX-M-15) [4, 17–19]. Similarly, as shown in Table 3, in Italian LTCFs the most prevalent ESBLs in *E. coli* were found to be CTX-M-type enzymes (79–97 %), particularly CTX-M-15, and these *E. coli* mainly belonged to the ST131 clonal group [14, 20–22]; in a study of isolates from 12 LTCFs the H30-ST131 subclone strongly predominated (71 %) [23]. On the other hand, ESBLs in *Klebsiella pneumoniae* are mainly encoded by *bla*_CTX-M_ or *bla*_SHV-12_ genes, whereas the ESBL phenotype of *Proteus mirabilis* and *Morganella morganii* isolates is generally due to production of the TEM-92 enzyme; high prevalence of the plasmidic AmpC-type enzyme CMY-16 was also found in *P. mirabilis* [14, 20, 22, 24, 25].

**Table 3:** Characteristics of MDR *Enterobacteriaceae* isolated from surveillance or clinical samples of Italian LTCF residents or staff

Italian Province, year	Bacterial species	Resistance gene (number of isolates)	Clonal group (number of isolates)	References
ESBL or high-level AmpC producing *Enterobacteriaceae*				
Pavia, 2003–04	*E. coli*	*bla*_*CTXM*_ (61)		[21]
		*bla*_*SHV-5*_ (1)		
		*bla*_*TEM*_ (15)		
Various provinces, 2006		*bla*_*CTXM*_ (41)		[22]
		*bla*_*SHV-5*_ (1)		
		*bla*_*SHV-2*_ (1)		
		AmpC (3)		
Lodi, 2009–10		*bla*_*CTXM*_ (61)		[20]
		*bla*_*SHV-type*_ (2)		
Bolzano, 2012		*bla*_*CTXM-15*_ (45)	ST131 (50)	[14]
		*bla*_*CTXM-9*_ (4)		
		*bla*_*SHV-5/12*_ (6)		
		*bla*_*TEM-type*_ (1)		
Various provinces, 2006	*K. pneumoniae*	*bla*_*CTXM*_ (6)		[22]
		*bla*_*TEM-151*_ (1)		
Lodi, 2009–10		*bla*_*CTXM-15*_ (11)		[20]
Bolzano, 2012		*bla*_*SHV-12*_ (9)		[14]
		*bla*_*CTXM-15*_ (1)		
Pavia, 2004–05	*P. mirabilis*	*bla*_*TEM-92*_ (23)		[25]
		*bla*_*CMY-16*_ (3)		
Pavia, 2003–06		ESBL (197)		[24]
		*bla*_*CMY-16*_ (20)		
Various provinces, 2006		*bla*_*TEM-92*_ (23)		[22]
		*bla*_*TEM-92 +*_ *bla*_*CMY-16*_ (4)		
		*bla*_*TEM-4*_ (9)		
		*bla*_*TEM-24*_ (1)		
Lodi, 2009–10		*bla*_*TEM-92*_ (10)		[20]
		*bla*_*CMY-16*_ (15)		
Bolzano, 2012		*bla*_*TEM-92*_ (17)		[14]
		*bla*_*CMY-16*_ (1)		
Various provinces, 2006	*M. morganii*	*bla*_*TEM-92*_ (14)		[22]
Bolzano, 2012		*bla*_*TEM-92*_ (10)		[14]
Various provinces, 2015	*E. coli*	Not reported	H30-ST131 (175)	[23]
Carbapenemase producing *Enterobacteriaceae*				
Bolzano, 2008–12	*E. coli*	*bla*_*VIM-1*_ (14)	Typing of 3 *E. coli*: all ST131; 1 *E. coli* subtyped: H30-ST131	[13, 14, 29, 30, 32]
	*K. pneumoniae*	*bla*_*VIM-1*_ (5)		
	*Klebsiella oxytoca*	*bla*_*VIM-1*_ (4)		
	*M. morganii*	*bla*_*VIM-1*_ (1)		
	*P. mirabilis*	*bla*_*VIM-1*_ (1)		
	*Providencia stuartii*	*bla*_*VIM-1*_ (1)		
Bologna, 2011	*K. pneumoniae*	*bla*_*NDM-1*_ (4)		[37]
Milan, 2011–13	*E. coli*	*bla*_*KPC-2*_ (9)	ST131 (8)	[33]
		*bla*_*KPC-3*_ (1)		
		*bla*_*KPC-8*_ (1)		
Cuneo, 2013	*E. coli*	*bla*_*VIM-1*_ (1)	H30- ST131 (1)	[32]
Valle d’Aosta, 2013–14	*K. pneumoniae*	*bla*_*KPC-2*_ (6)	ST1789 (6)	[36]
Various provinces, 2015	*K. pneumoniae*	*bla*_*KPC-3*_ (3)		[23]
	*E. coli*	*bla*_*VIM-1*_ (2)		

Sporadic or epidemic carbapenemase producing *Enterobacteriaceae* have been reported from European LTCF residents: *K. pneumoniae* expressing the carbapenemase OXA-48 from the Netherlands [5], *E. coli* and *K. pneumoniae* producing *K. pneumoniae* carbapenemase 2 (KPC-2) from Greece [26], *K. pneumoniae* producing KPC-3 from Portugal [27] and *K. pneumoniae* producing New Delhi metallo-β-lactamase-1 (NDM-1) from Poland [28]. The prevalent carbapenemase-types in enterobacterial isolates from Italian LTCFs are the Verona integron-encoded metallo-β-lactamase-1 (VIM-1) and various KPC types (Table 3). VIM-1 carbapenemases have been found to be expressed from various species of *Enterobacteriaceae*, isolated from acute-care hospitals and LTCFs in the Province of Bolzano [29, 30]; in this setting the *bla*_VIM-1_ gene is located on plasmids of various sizes, but with the same framework and all belonging to the incompatibility group IncN [31]. In Italian LTCFs VIM-1 enzymes have been found to be associated with *E. coli* belonging to the clonal group ST131 [14, 29]; subtyping of 2 isolates identified them as the subclone H30 ST131 [32]. Moreover, in a long-term care and rehabilitation facility in Milan an outbreak caused by an ST131 *E. coli* strain, associated with *bla*_KPC-2_ and *bla*_KPC-8_ genes located on plasmids belonging to the IncF group, has been identified [33]. KPC-producing *Enterobacteriaceae* are widely distributed in LTCFs of countries like the USA with high prevalence of this resistance phenotype [34]. KPC-producing *K. pneumoniae* are epidemically diffused in Italy, mostly belonging to the clonal complex 258 [35], yet in a study involving 489 residents from 12 LTCFs only 5 isolates were found to produce carbapenemases (1.0 % of residents colonized); of these isolates 3 *K. pneumoniae* harbored *bla*_KPC-3_ and 2 *E. coli* carried *bla*_VIM-1_ [23]. Further sporadic KPC-2 producing *K. pneumoniae* isolates from LTCF residents were found by other authors [36]. Finally, an outbreak of NDM-1 producing *K. pneumoniae* in a nursing home in Northern Italy has been identified [37].

Finally, the emergence of the transferable colistin resistance determinant *mcr-1* has most recently been reported in commensal *E. coli* isolates from three Italian LTCFs [38].

MRSA isolates from an Italian LTCF were all negative for Panton-Valentine leukocidin (PVL) and belonged prevalently to ST8, *spa* t008, *SCCmec* IV [39]; this MRSA type is also prevalent in isolates from acute care hospitals in Northern Italy [40, 41]. In LTCF residents of other European countries various predominant MRSA types have been found: ST22, *spa* t022 or t032 (EMRSA-15) in the United Kingdom [42–44] and Ireland [9], the same MRSA type together with ST5, *spa* t003 (EMRSA-3) in Germany [45, 46], ST45, *SCCmec* IV in Belgium [47] and ST146, *SCCmec* IV in Spain [48]. Livestock-associated MRSA (LA-MRSA) genotypes belonging to CC398 have also sporadically been found in European LTCFs [49], whereas the isolation of PVL producing MRSA isolates has not yet been reported [9, 43, 44, 48].

### Risk factors for colonization by MDR organisms of residents in Italian and other European LTCFs

Residents of LTCFs are uniquely vulnerable to colonization by MDR organisms and infection with these bacteria, because of the frequent and simultaneous presence of major risk factors [1, 50]; various of these risk factors have been found in screening studies in Italy and other European countries (Table 4). Particularly, the previous administration of antibiotics, especially fluoroquinolones and extended-spectrum cephalosporins, the presence of invasive medical devices, older age and the degree of dependence on nursing care, expressed as physical disability, are associated with colonization by MDR organisms. A striking feature is the high colonization of LTCF staff with MDR bacteria; this carriage probably reflects resident-to-staff, and, perhaps, staff-to-staff transmission [13]. LTCF residents frequently depend on continuous nursing care for daily living activities, with many occasions for horizontal transmission of MDR-organisms between residents and health care workers (resident-to-staff and staff-to-resident transmission). The length of stay in a LTCF is also a major risk factor, suggested by the higher colonization rates in LTCF residents compared with acute care hospital patients [13]. Moreover, the strict application of hospital hygiene measures in LTCFs is difficult because they are homes of the residents and therefore social interactions are encouraged. Previous hospitalization of LTCF residents is a further risk factor for colonization with MDR bacteria because patients can be admitted to the LTCF already colonized with MDR organisms acquired in the hospital [1]; surgical procedures and various chronic diseases and medications may increase the frequency of contact of LTCF residents with the health care system and facilitate the colonization and infection with MDR organisms. Interestingly, in a Dutch study authors found that the presence of MRSA carriers in the LTCFs more than doubled the likelihood of finding ESBL-producing *E. coli* [51]; this might be explained by similar transmission ways of these MDR organisms among LTCF residents. Finally, several further facility specific risk factors have been described, comprising lack of infection control policy, inadequate staffing and high staff turnover, increased number of residents per bedroom and the presence of limited facilities for hand washing [1].

**Table 4:** Risk factors for colonization of Italian and other European LTCF residents with ESBL producing *Enterobacteriaceae* and/or MRSA

Risk factor	Bacterial resistance phenotype	References (Italy)	References (Other European countries)
High age	ESBL, MRSA	[13]	[8, 9, 60]
Male sex	ESBL, MRSA		[6, 9, 42, 46]
Physical disability, bedridden, low functional status	ESBL, MRSA	[13, 23]	[6, 8, 45–47, 49, 59–61]
Prolonged duration of stay in LTCF	MRSA	[23]	
Invasive medical devices (urinary catheter, percutaneous enteral gastrostomy tube, tracheostomy tube)	ESBL, MRSA	[13, 20]	[7, 8, 42, 45, 46, 52, 59, 60]
Previous administration of antibiotics within the preceding year	ESBL, MRSA	[13, 20, 62]	[6, 7, 17, 47–49, 51, 52, 54, 58–61]
Colonization history by the same microorganism	ESBL, MRSA		[6, 7, 46, 49, 59, 61]
MDR bacteria carriage of other residents	MRSA		[46, 51]
Multiple room occupancy, MDR carrier in the same room	MRSA		[46]
Residency in specific unit within LTCF	ESBL, MRSA	[13, 14, 62]	
Prior admission to acute care hospital	ESBL, MRSA	[20, 62]	[42, 45–47, 58, 60, 61]
Surgical procedures within 30 days	ESBL	[20]	
Cancer	ESBL, MRSA	[13, 62]	[58]
Decubitus ulcer	ESBL, MRSA	[22]	[46, 48, 59, 60]
Various wounds, skin lesions	MRSA, ESBL		[6, 8, 45–47, 59]
Peptic ulcer, use of antiacids	ESBL, MRSA	[20]	[6]
Chronic renal failure	ESBL	[20]	
History of urinary tract infections	ESBL		[17]
Urinary and faecal incontinency	ESBL, MRSA	[23]	[8, 58]
Chronic obstructive pulmonary disease (COPD)	ESBL, MRSA	[13, 20]	
Diabetes	ESBL	[20]	[8]

## Conclusions

LTCFs are important reservoirs for MDR organisms and colonization rates are similar or even higher than those found in acute care hospitals [13, 14], but the great variability in the number of residents and staff members and in the methods used for screening make it difficult to directly compare the results of various colonization studies. Nevertheless, colonization prevalence with MDR bacteria of LTCF residents has been found to be highly variable, reaching values as high as 37.6 % in France for MRSA [52] and as high as 55-75 % in Ireland for ESBL producing *Enterobacteriaceae* [4, 17]. In the few published surveillance studies in Italian LTCF residents similarly high ESBL colonization rates have been reported, whereas MRSA colonization was lower. Especially worrying is the emergence of carbapenemase producers in these facilities, expanding the reservoir of this health care threat. Colonization is frequently the prerequisite for infections [53]; this has many implications with regard to hospital hygiene measures and therefore reinforced infection control and surveillance programs are urgently needed, though their application in this specific setting is challenging [1]. Engaging of LTCFs in antimicrobial stewardship programs, frequently set up in acute care hospitals, is also a critical “hot topic”, focusing on drivers for antimicrobial over-use [2].

Data referred to baseline colonization by MDR bacteria in the Italian LTCF setting is not representative of the whole country and therefore the performance of proper countrywide screening studies is required; moreover, a standardized screening protocol is recommended. For this reason and to promote further studies of various microbiological aspects related to LTCFs, the Association of Italian Clinical Microbiologists (Associazione Microbiologi Clinici Italiani; AMCLI) in 2016 has set up a new Working Group for the Study of Infections in LTCFs (Gruppo di Lavoro per lo Studio delle Infezioni nelle Residenze Sanitarie Assistite﻿﻿﻿ e Strutture Territoriali assimilabili; GLISTer), consisting of Clinical Microbiologists represented by the authors of this review article. The mission of this group is also to support infection prevention and control initiatives in Italian LTCFs, with the aim to reduce the spread of MDR bacteria within and across acute and chronic health care facilities.

## Abbreviations

AMCLI: Associazione Microbiologi Clinici Italiani
AmpC: Group of cephalosporinases
Bla: β-lactamase
CC: Clonal complex
CMY: Cephalosporinase type
CPE: Carbapenemase-producing *Enterobacteriaceae*
CTX-M: Cefotaximase-Munich, ESBL type
ESBL: Extended-spectrum β-lactamase
GLISTer: Gruppo di Lavoro per lo studio delle Infezioni nelle Residenze Sanitarie Assistite e Strutture Territoriali assimilabili
Inc: Incompatibility-group
KPC: *Klebsiella pneumoniae* carbapenemase
LTCF: Long-term care facility
mcr: Colistin resistance determinant
MDR: Multi-drug resistant
MRSA: Methicillin-resistant *Staphylococcus aureus*
NDM-1: New Delhi metallo-β-lactamase
OXA-48: Type of carbapenemase, OXA stays for oxacillinase
PVL: Panton-Valentine leukocidin
*SCCmec*: Staphylococcal chromosome cassette *mec*
SHV: β-lactamase type, SHV stays for sulfhydryl-variable
*spa*: Staphylococcal protein A type
ST: Sequence type
TEM: β-lactamase type, TEM stays for the Greek patient name “Temoneira”
VIM: Integron-encoded metallo-β-lactamase
VRE: Vancomycin-resistant enterococci
